# The generation and analysis of a novel combination of recombinant adenovirus vaccines targeting three tumor antigens as an immunotherapeutic

**DOI:** 10.1186/2051-1426-3-S2-P452

**Published:** 2015-11-04

**Authors:** Kwong Y Tsang, Elizabeth S Gabitzsch, Claudia Palena, Justin M David, Massimo Fantini, Anna R Kwilas, Adrian E Rice, Yvette Latchman, James W Hodge, Caroline Jochems, Romaine I Fernando, James Gulley, Ravi A Madan, Christopher R Heery, Joseph P Balint, Frank R Jones, Jeffrey Schlom

**Affiliations:** 1 National Cancer Institute, National Institutes of Health, Bethesda, MD, USA; 2 Etubics Corporation, Seattle, WA, USA; 3 Laboratory of Tumor Immunology and Biology, Center for Cancer Research, National Cancer Institute, National Institutes of Health, Bethesda, MD, USA; 4 Genitourinary Malignancies Branch, National Cancer Institute, National Institutes of Health, Bethesda, MD, USA

## 

We have reported on a novel adenovirus serotype 5 (Ad5) vector gene delivery platform (Ad5 [E1-, E2b-]), in which regions of the early 1 (E1), early 2 (E2b), and early 3 (E3) genes have been deleted. The unique deletions in this platform result in a dramatic decrease in late gene expression, leading to a marked reduction in host immune response to the vector. CEA, MUC1, and brachyury are tumor-associated antigens (TAA) expressed on a wide range of human tumors. Ad5 [E1-, E2b-]–CEA vaccine (ETBX-011) has been employed in clinical studies as an active vaccine to induce immune responses to CEA in metastatic colorectal cancer patients. The Ad5 [E1-, E2b-]–CEA vector encodes the entire CEA sequence modified to express an enhancer T-cell epitope. We report here the development of novel Ad5 [E1-, E2b-]–brachyury and Ad5 [E1-, E2b-]–MUC-1 vaccine constructs. The Ad5 [E1-, E2b-]–brachyury vector was constructed to encode the entire brachyury gene devoid of 25 amino acids involved in DNA binding, and modified to express an enhancer T cell epitope. The Ad5 [E1-, E2b-]–MUC-1 vector was constructed to encode the entire MUC-1 transgene with eight agonist epitopes, including five in the C-terminus. Our results show that these constructs (CEA, MUC1 and brachyury) are capable of activation, as well as generation of antigen specific T cells *in vitro*, and of inducing antigen-specific T cells in vaccinated mice. We have also demonstrated that the use of a combination of the three vaccines (designated Tri-Ad5) displays little, if any, antigenic competition in *in vitro* studies of human dendritic cells for antigen-specific T cell activation and generation, or in murine vaccination studies. The studies reported here support the rationale for the application of Tri-Ad5 as a therapeutic modality to induce immune responses to a diverse range of human TAAs for potential clinical studies.

